# BKM120 sensitizes glioblastoma to the PARP inhibitor rucaparib by suppressing homologous recombination repair

**DOI:** 10.1038/s41419-021-03805-6

**Published:** 2021-05-26

**Authors:** Shaolu Zhang, Xin Peng, Xiaofei Li, Hongyan Liu, Baoquan Zhao, Moshe Elkabets, Yao Liu, Wei Wang, Ran Wang, Yuxu Zhong, Dexin Kong

**Affiliations:** 1 Tianjin Key Laboratory on Technologies Enabling Development of Clinical Therapeutics and Diagnostics, School of Pharmacy, Tianjin Medical University, Tianjin, China; 2 State Key Laboratory of Toxicology and Medical Countermeasures, Beijing Institute of Pharmacology and Toxicology, Beijing, China; 3 The Shraga Segal Department of Microbiology, Immunology and Genetics, Faculty of Health Sciences, Ben-Gurion University of the Negev, Beer-Sheva, Israel; 4 Department of Otorhinolaryngology Head and Neck, Institute of Otorhinolaryngology, Tianjin First Central Hospital, Tianjin, China; 5 School of Medicine, Tianjin Tianshi College, Tianyuan University, Tianjin, China

**Keywords:** Targeted therapies, Drug development

## Abstract

PARP inhibitors have been approved for the therapy of cancers with homologous recombination (HR) deficiency based on the concept of “synthetic lethality”. However, glioblastoma (GBM) patients have gained little benefit from PARP inhibitors due to a lack of BRCA mutations. Herein, we demonstrated that concurrent treatment with the PARP inhibitor rucaparib and the PI3K inhibitor BKM120 showed synergetic anticancer effects on GBM U251 and U87MG cells. Mechanistically, BKM120 decreased expression of HR molecules, including RAD51 and BRCA1/2, and reduced HR repair efficiency in GBM cells, therefore increasing levels of apoptosis induced by rucaparib. Furthermore, we discovered that the two compounds complemented each other in DNA damage response and drug accumulation. Notably, in the zebrafish U87MG-RFP orthotopic xenograft model, nude mouse U87MG subcutaneous xenograft model and U87MG-Luc orthotopic xenograft model, combination showed obviously increased antitumor efficacy compared to each monotherapy. Immunohistochemical analysis of tumor tissues indicated that the combination obviously reduced expression of HR repair molecules and increased the DNA damage biomarker γ-H2AX, consistent with the in vitro results. Collectively, our findings provide new insight into combined blockade of PI3K and PARP, which might represent a promising therapeutic approach for GBM.

## Introduction

Glioblastoma (GBM), the most malignant tumor of the adult central nervous system, represents up to 50% of all primary brain gliomas^1^. Current GBM therapy, including aggressive surgical resection, high-dose external beam radiation therapy (RT), and temozolomide (TMZ) chemotherapy, is only associated with a median time to progression of 6 months and a median overall survival of 15 months^2,3^. Therefore, novel therapies for GBM are still urgently needed.

Poly (ADP-ribose) polymerase (PARP) is a nuclear protein known to function as a DNA damage sensor and plays a role in DNA repair pathways via poly-ADP-ribosylating the automodification domain and enabling the recruitment of DNA repair proteins^2,4^. PARP inhibition ultimately causes double-strand break (DSB) accumulation during DNA replication and induces apoptosis, particularly in cells with homologous recombination (HR) deficiency. This is the basis for the synthetic lethality of PARP inhibitors (PARPis) in cancers with HR deficiency due to mutations in BRCA1/2 or other HR genes^5^, allowing these cancer cells to be selectively targeted while sparing normal cells that have intact DNA repair systems^6^. Most recently, targeting autophagy was also demonstrated to enhance the therapeutic efficacy of PARP inhibitor in HR proficient breast cancer cells^7^. Several PARPis have been approved for the therapy of HR-deficient tumors^8,9^. However, since GBM is known to lack BRCA1/2 mutations and is, therefore, HR proficient, PARPi alone might not exhibit optimal antitumor efficacy based on the synthetic lethality theory. The efficacy of PARPi was reported to be limited due to the superactivation of the PI3K-AKT signaling pathway^10,11^, and loss of its counterpart PI3K-PTEN was found in approximately 36% of GBMs^11^. Therefore, PI3K inhibitors (PI3Kis) are expected to perform well in combination with PARPi to treat GBM.

BKM120 is a pan-PI3Ki in clinical trials for cancer therapy^12^. Until now, pan-PI3Kis, including BKM120, have shown modest benefits in the clinic because their doses for therapy are limited due to toxicity^13^. Since drug combinations may enhance efficacy while minimizing toxicity, we recently examined the antitumor effect of the combination of PI3Ki BKM120 and the PARPi rucaparib on GBM in vitro and in vivo. We verified that BKM120 induced HR deficiency by downregulating the DNA repair process and sensitized BRCA-proficient tumor GBM to PARP inhibition, providing novel insights into the treatment of GBM.

## Results

### Combination of BKM120 and rucaparib exhibits a synergetic antiproliferative effect on GBM U251 and U87MG cells

**Fig. 1:**
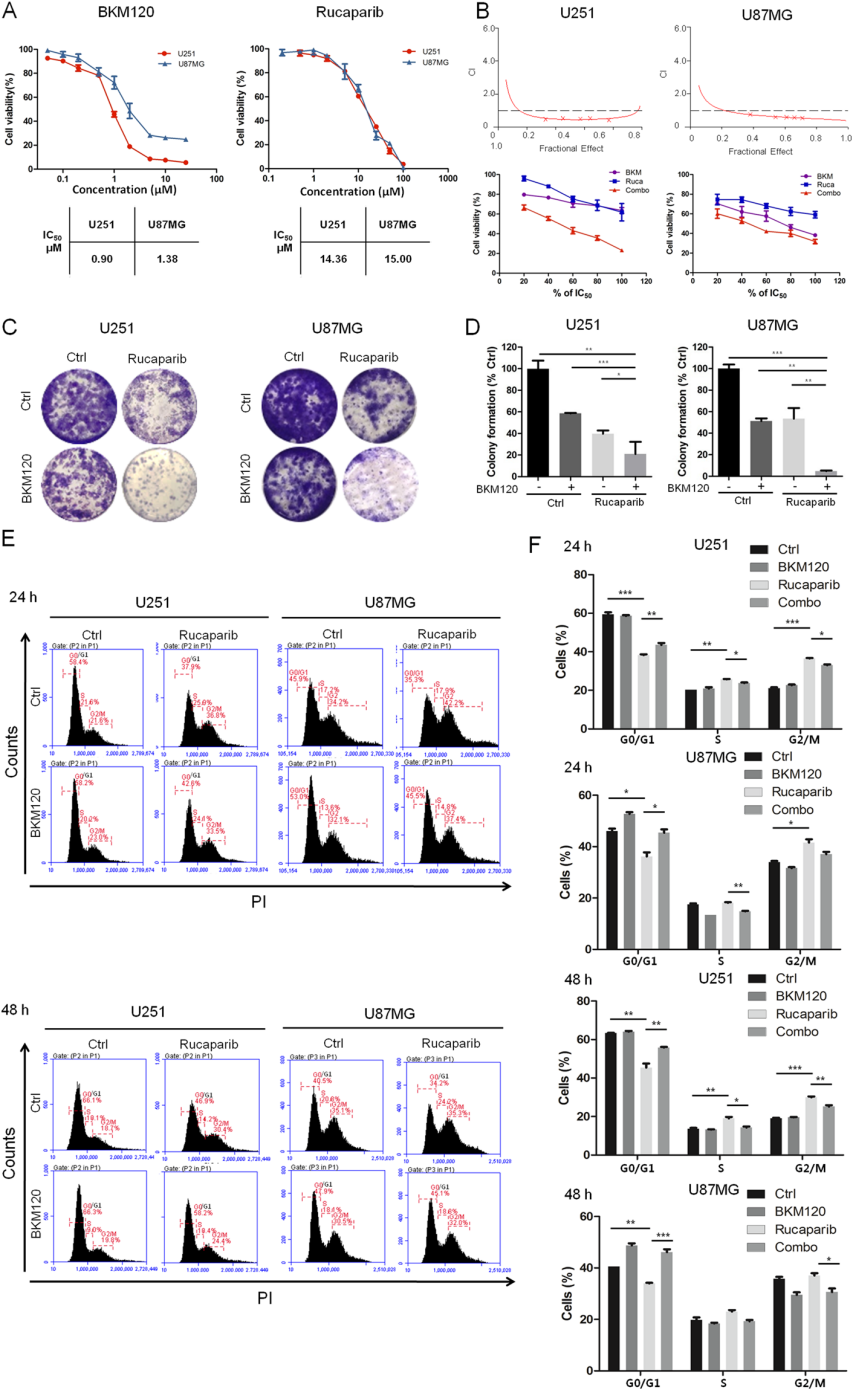
The in vitro antiproliferative activities of BKM120 and/or rucaparib in human GBM cells. **A** U251 and U87MG cells were treated with various concentrations of BKM120 and rucaparib (BKM-120 0.05, 0.1, 0.2, 0.5, 1, 2, 5, 10, 25 μM; rucaparib-0.2, 0.5, 1, 2, 5, 10, 25, 50, 100 μM), and antiproliferative effects were determined by MTT assay following 96 h incubation. **B** Cells were treated with BKM120 and rucaparib as single agents or in combination for 96 h and then subjected to MTT assay. The drug combination effects were analyzed using CalcuSyn software, and the resulting CI-Fa plots are shown. **C** U251 cells were treated with 0.5 μM BKM120 or/and 10 μM rucaparib, and U87 cells were treated with 1 μM BKM120 or/and 10 μM rucaparib (the same concentrations were used in subsequent experiments unless indicated) for 10 days and then stained with crystal violet. **D** Colonies with 50 or more cells were quantified using ImageJ. **E** Cells were treated with BKM120 and/or rucaparib for 24/48 h. After PI staining, flow cytometry analysis was performed to determine the cell cycle distribution. **F** Cell cycle distribution of the cells exposed to the single drugs or combination are represented as stacked columns. Statistical analysis of G2/M phase in each group. All data are presented as the mean ± SD (*n* = 3). *P < 0.05; **P < 0.01; ***P < 0.001.

We first examined the antiproliferative effect of BKM120 and rucaparib using an MTT assay. As shown in Fig. 1A, either of the two compounds alone inhibited the proliferation of GBM U251 and U87MG cancer cells in a dose-dependent manner, with IC_50_ values of 0.90 and 1.38 μM (BKM120) and 14.36 and 15.00 μM (rucaparib), respectively. Next, we determined the antiproliferative effect of the combination of BKM120 and rucaparib (with ratios of 0.5 vs 10 μM for U251 cells and 1 vs 10 μM for U87MG cells, the same as the following in vitro experiments) and analyzed their combinational effect using Chou-Talalay’s method^14^. As indicated in Fig. 1B and Tables 1 and 2, all the combination index (CI) values were less than 1, suggesting that the combinations were synergetic. Next, we performed colony formation assays to determine the effect of the combinations on the tumorigenic ability of GBM cells. The colony formation of U251 and U87MG cells with a higher potency than either drug alone (Fig. 1C, D). As cell cycle progression is essential for cell proliferation, we investigated the effect of this treatment combination on the cell cycle by flow cytometry analysis. Figure 1E, F and Fig. S1 indicated that treatment with rucaparib for 24/48/72 h led to G2/M cell cycle arrest, but the effect was attenuated when treated in the presence of BKM120.

**Table 1:** Combination indexes (CI) of BKM120 and Rucaparib in U251 cells.

Product	Gene target	Indication	Route	Clinical trial identifier(s)
**ASOs**				
Tominersen (Ionis Pharmaceuticals/Roche)	Huntingtin	Huntington’s disease	Intrathecal	NCT03761849
Tofersen, BIIB067 (Ionis Pharmaceuticals/Biogen)	SOD-1	Amyotrophic lateral sclerosis	Intrathecal	NCT02623699
**GalNAc−siRNA conjugates**				
Fitusiran, ALN-AT3sc (Sanofi/Alnylam Pharmaceuticals)	SERPINC1	Haemophilia and rare bleeding disorders	Subcutaneous	NCT03417102, NCT03417245, NCT03549871, NCT03754790
Vutrisiran, ALN-TTRsc02 (Alnylam Pharmaceuticals)	TTR	TTR-mediated amyloidosis	Subcutaneous	NCT03759379
**GalNAc−ASO conjugates**				
AKCEA-TTR-L_Rx_ (Akcea/Ionis Pharmaceuticals)	TTR	TTR-mediated amyloid polyneuropathy or cardiomyopathy	Subcutaneous	NCT04136184, NCT04136171
Pelacarsen, AKCEA-APO(a)-L_Rx_ (Akcea/Ionis Pharmaceuticals)	Apoliprotein A1	Cardiovascular disease	Subcutaneous	NCT04023552
Nedosiran, DCR-PHXC (Dicerna Pharmaceuticals)	Lactate dehydrogenase	Primary hyperoxaluria	Subcutaneous	NCT04042402
**LNPs**				
Onpattro, patisiran (Alnylam Pharmaceuticals)	TTR siRNA	Cardiomyopathy-associated TTR-mediated amyloidosis	Intravenous	NCT03997383
**AAV vectors**				
Lumevoq, lenadogene Nolparvovec, GS010 (GenSight Biologics)	ND4 (AAV2)	Leber hereditary optic neuropathy	Intravitreal	NCT03293524, NCT02652767, NCT02652780, NCT03153293
AVXS-101 (Novartis)	SMN2 (AAV9)	Spinal muscular atrophy	Intrathecal	NCT03505099, NCT03461289, NCT03306277, NCT03837184
Valoctocogene roxaparvovec, BMN 270 (BioMarin Pharmaceutical)	FVIII (AAV5)	Haemophilia A	Intravenous	NCT03370913, NCT04323098, NCT03392974
Etranacogene dezaparvovec AMT-061 (uniQure)	FIX-Padua (AAV5)	Haemophilia B	Intravenous	NCT03569891
Fidanacogene elaparvovec, SPK-9001, (Pfizer, Spark Therapeutics)	FIX-Padua (AAV100)	Haemophilia B	Intravenous	NCT03587116
Timrepigene emparvovec, NSR-REP1 (Biogen/Nightstar Therapeutics)	REP1 (AAV2)	Chloridaemia	Subretinal	NCT03496012
Olenasufligene relduparvovec, LYS-SAF302 (Lysogene)	SGSH (AAVrh10)	MPS-IIIA	Intracerebral	NCT03612869
NSR-RPGR (Biogen/Nightstar Therapeutics)	RPGR (AAV8)	X-linked retinitis pigmentosa	Subretinal	NCT03116113
FLT180a (Freeline Therapeutics)	FIX (AAVs3)	Haemophilia B	Intravenous	NCT03641703
**Adenovirus serotype vectors**				
Sputnik V, Gam-Covid-Vac (Gamaleya Research Institute)	SARS-CoV-2 spike protein (Ad26/Ad5)	COVID-19	Intramuscular	NCT04530396

**Table 2:** Combination indexes (CI) of BKM120 and Rucaparib in U87MG cells.

Drug or drug combination	*r*	CI values		
		ED50	ED75	ED90
BKM120 (1 μM)	0.95250		—	—
Rucaparib (10 μM)	0.93404	—	—	—
Combo	0.99831	0.66068	0.53476	0.46661

### Combination of rucaparib with BKM120 enhances apoptosis and DNA damage in GBM U251 and U87MG cells

**Fig. 2:**
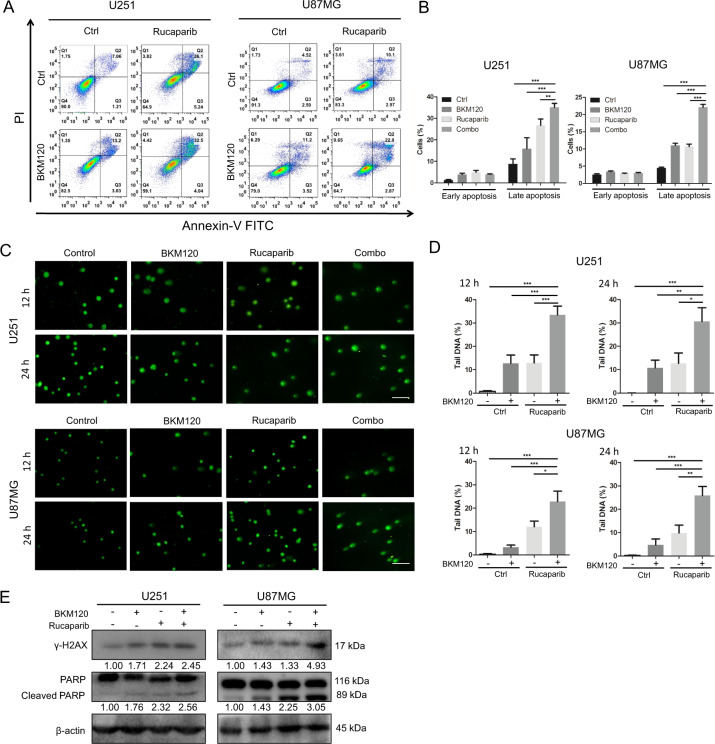
BKM120 and rucaparib cooperate to induce apoptosis and DNA damage in GBM cells. U251 and U87MG cells were treated with BKM120 and rucaparib as single agents or in combination. **A** The percentage of apoptotic cells was determined by Annexin V and PI staining at 72 h. **B** FACS quantification of the respective cell population including Annexin V + /propidium iodide− early apoptotic cells, and Annexin V + /propidium iodide+ late apoptotic cells. **C** DNA damage in GBM cells was determined by alkaline comet assay to detect both SSBs and DSBs for 12 and 24 h. Comet images × 200 acquired by fluorescence microscopy are shown. Scale bar: 100 μm. **D** The tail moment was used to quantify DNA damage with CASP software. **E** The expression levels of the apoptosis marker PARP (cleaved and full length) and the DNA DSB marker γ-H2AX were determined by western blotting at 48 h. All data are presented as the mean ± SD (*n* = 3). *P < 0.05; **P < 0.01; ***P < 0.001.

To determine the effect of the combination on apoptosis in GBM cells, we next conducted Annexin V-PI staining and flow cytometry analysis. Compared to each drug alone, the combined treatment led to a significant increase in the late apoptotic population in U251 and U87MG cells for 24 and 72 h (Fig. 2A, B and Suppl Fig. 2). Since DNA damage plays a key role in apoptosis, we next determined whether the drug combination enhanced DNA damage using an alkaline comet assay for detection of both SSBs (single strand breaks) and DSBs in DNA. As shown in Fig. 2C, D, compared to each single-agent treatment, combination of the 2 drugs generated markedly increased tail intensity in U251 and U87MG cells, suggesting more severe DNA damage. Consistently, combination treatment induced a dose-dependent increase in the apoptosis marker cleaved PARP and the DNA DSB marker γ-H2AX (Fig. 2E).

### Combination of rucaparib with BKM120 reduces HR repair in GBM U251 and U87MG cells

**Fig. 3:**
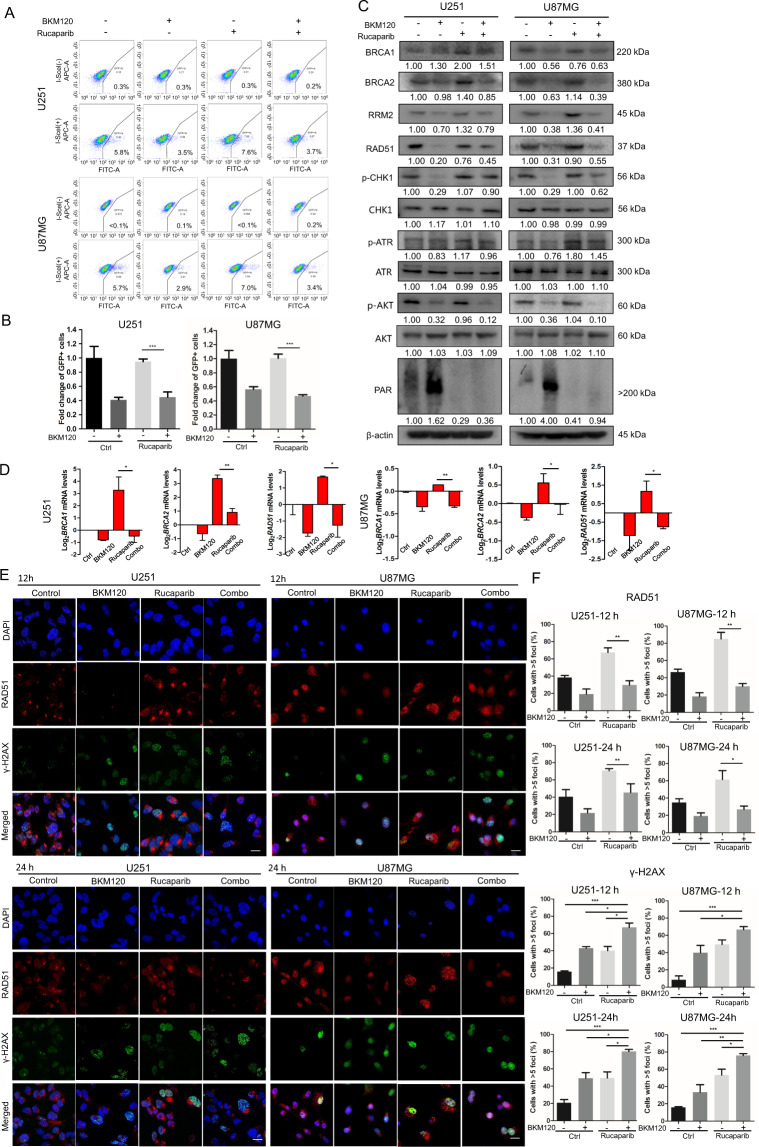
Effects of BKM120 and rucaparib as single agents or in combination on HR repair. **A** U251 and U87MG cells were transfected with DR-GFP plasmid and pCBASceI plasmid using Lipofectamine 3000. Cells were treated with BKM120 or/and rucaparib for 72 h and then collected and resuspended in ice-cold PBS. GFP intensity was analyzed by flow cytometry. **B** The percentage of GFP-positive cells represents HR repair efficiency. **C**, **D** U251 and U87MG cells were treated with BKM120 and/or rucaparib for 48 h. **C** Cells were collected for western blotting detection of DNA damage- and HR repair-related proteins, including BRCA1, BRCA2, RAD51, RRM2, p-ATR, p-CHK1, p-AKT, ATR, CHK1, and PAR. **D** Quantitative reverse transcription PCR analysis of BRCA1, BRCA2, and RAD51 expression in two cancer cell lines. Gene expression was normalized to 18 s rRNA. **E**, **F** Cells were treated with BKM120 or/and Rucaparib for 12/24 h. Representative images of immunofluorescence staining of RAD51 and γ-H2AX in GBM cells Cell nuclei were stained with DAPI. Scale bar: 20 μm. The percentage of cells with RAD51/γ-H2AX foci was shown in (**F**). All data are presented as mean ± SD (*n* = 3). *P < 0.05; **P < 0.01; ***P < 0.001.

Since HR plays a key role in the repair process of DNA DSB damage, we next investigated the effect of the combination on HR repair. First, an HR reporter assay showed that HR repair efficiency was reduced after cotreatment of rucaparib with BKM120 (Fig. 3A, B). Compared to rucaparib alone, cotreatment with BKM120 reduced expression of the HR repair molecules BRCA1, BRCA2, and RAD51 at both the protein and mRNA levels (Fig. 3C, D). Consistently, immunofluorescence analysis indicated that the number of RAD51 foci was significantly reduced while the number of γ-H2AX foci was increased in U251 and U87MG cells in the combination of the 2 drugs compared to each drug alone (Fig. 3E, F). In addition, rucaparib increased expression of RRM2 (Ribonucleotide Reductase small subunit M2) and phosphorylation of ATR and CHK1 (checkpoint kinase 1), and these phenomena were reversed by cotreatment with BKM120. On the other hand, BKM120 alone induced increased poly-ADP-ribosylation (PAR), which was reversed by cotreatment with the PARPi rucaparib (Fig. 3C).

### BKM120 enhances the antitumor efficacy of rucaparib in a zebrafish U87MG orthotopic xenograft model

**Fig. 4:**
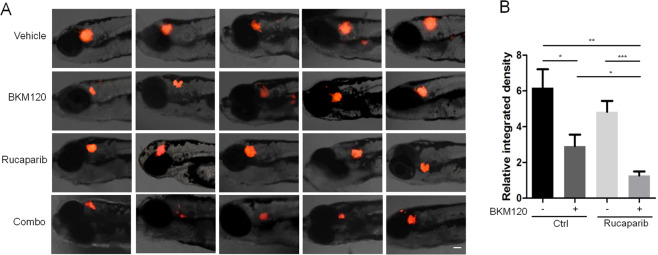
BKM120/rucaparib combination treatment inhibits tumor growth in the U87MG zebrafish xenograft model. **A** After transplantation with 50–100 U87MG-RFP cells (red fluorescence), the injected embryos were transferred to a 96-well plate containing drug and incubated for 96 h. The embryos were imaged with a fluorescence microscope to detect tumor growth. Scale bar: 100 μm. **B** The relative increase in fluorescence intensity was determined by dividing the measured values by the fluorescence intensity of the tumor in the same embryo at 0 days after implantation. Student’s *t*-test was used to compare the effect of each treatment with vehicle or treated animals. *P < 0.05; **P < 0.01; ***P < 0.001.

To evaluate the in vivo anti-GBM efficacy of the combination, we first determined the effect on a zebrafish U87MG orthotopic xenograft model. Tumor burden was evaluated by measuring the integrated RFP fluorescence intensity. As shown in Fig. 4A, B, BKM120 alone led to an ~50% reduction compared to the vehicle group. When combined with rucaparib, tumor growth was further inhibited, with an ~80% reduction compared to the vehicle group.

### BKM120 enhances the antitumor efficacy of rucaparib in a nude mouse U87MG subcutaneous xenograft model

**Fig. 5:**
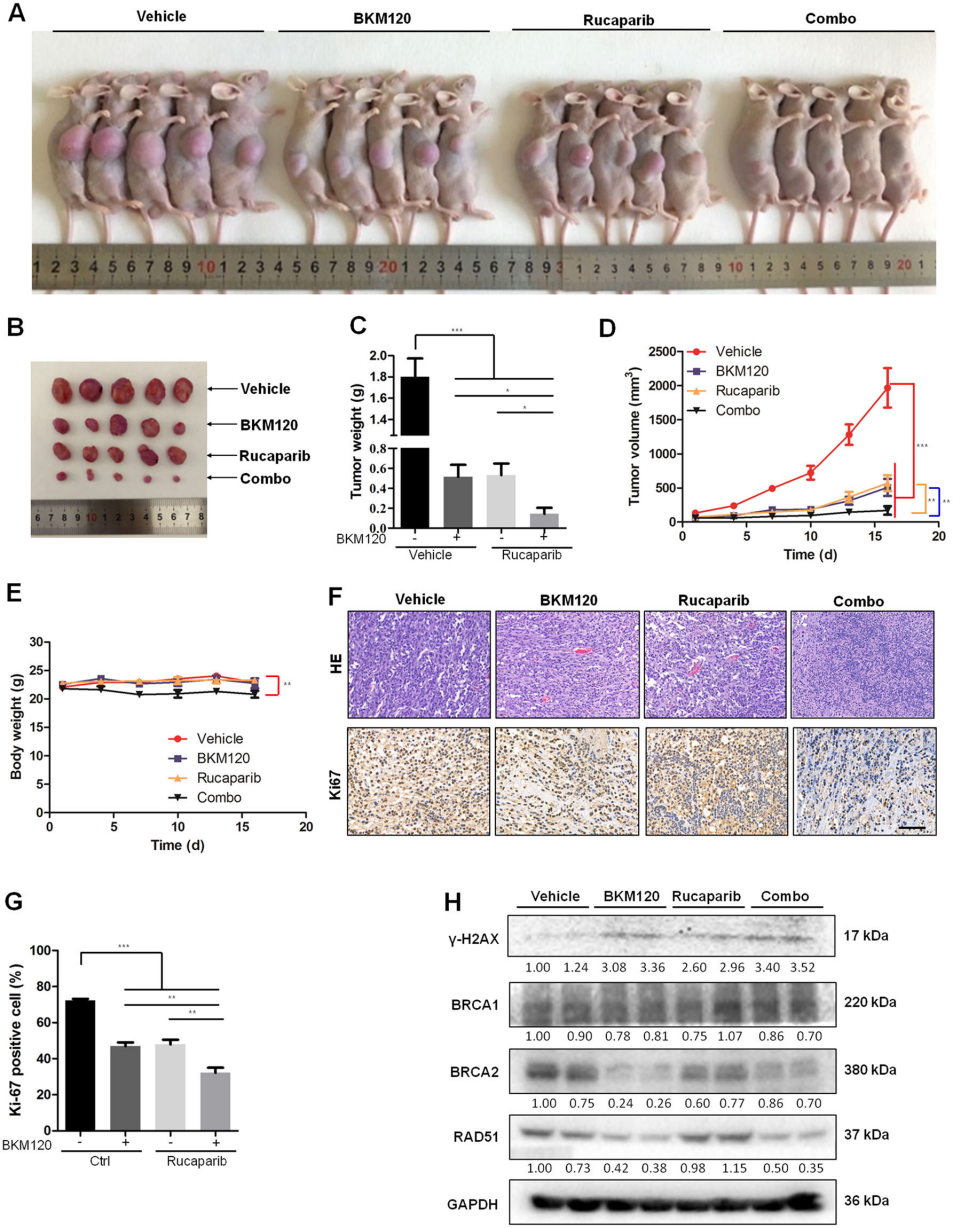
BKM120/rucaparib combination treatment markedly reduced tumor growth in U87MG nude mouse heterotopic xenograft models. **A**–**H** Nude mice with subcutaneous U87MG tumors were treated with vehicle, BKM120 (15 mg/kg), rucaparib (4 mg/kg), or the BKM120/rucaparib combination for 16 days (*n* = 7). After sacrificing mice, images were taken as shown in (**A**). Then, tumors were taken from mice with the pictures indicated in (**B**). **C** Measurement of tumor weight. Time-course measurements of tumor volumes (**D**) and body weights (**E**) every 3 days. **F** H&E and Ki67 immunohistochemistry staining of tumor tissues from U87MG xenograft mice treated with BKM120 and/or rucaparib. Scale bar: 50 μm. **G** Statistical quantification of (**F**) shows U87MG cell proliferation in xenografts with the indicated treatments. **H** western blotting analysis of BRCA1 and γ-H2AX in harvested tumors. Data are presented as the mean ± SD (*n* = 3). *P < 0.05; **P < 0.01; ***P < 0.001.

We subsequently investigated the antitumor efficacy of the drug combination using a nude mouse U87MG subcutaneous xenograft model. BKM120, rucaparib, the combination, and the vehicle were administered to the animal models. Tumor volume and body weight were measured every 3 days. Sixteen days later, mice were sacrificed, and tumors were excised for tumor weight measurement. As indicated in Fig. 5A–D, treatment with BKM120 or rucaparib as single agents led to robust inhibition of tumor growth both in volume and in weight. When administered in combination, tumor growth was further inhibited compared to each drug alone. By the end of the 16-day treatment period, the combination led to an over 90% reduction in both tumor volume and tumor weight. However, the combination also caused a slight decrease in body weight compared to each drug alone and to the vehicle group (Fig. 5E). Tumor tissues of each group were used for H&E, immunostaining and western blotting. As shown in Fig. 5F, G, the number of cells stained with Ki67, a biomarker of cell proliferation, was reduced after treatment with each drug alone or in combination, and the combination further inhibited U87MG proliferation compared to each drug alone. Western blot analysis showed that expression of γ-H2AX was increased, while BRCA1/2 and RAD51 was decreased after administration of the combination, consistent with the in vitro results (Fig. 5H).

### BKM120 enhances the antitumor efficacy of rucaparib in a nude mouse U87MG orthotopic xenograft model

**Fig. 6:**
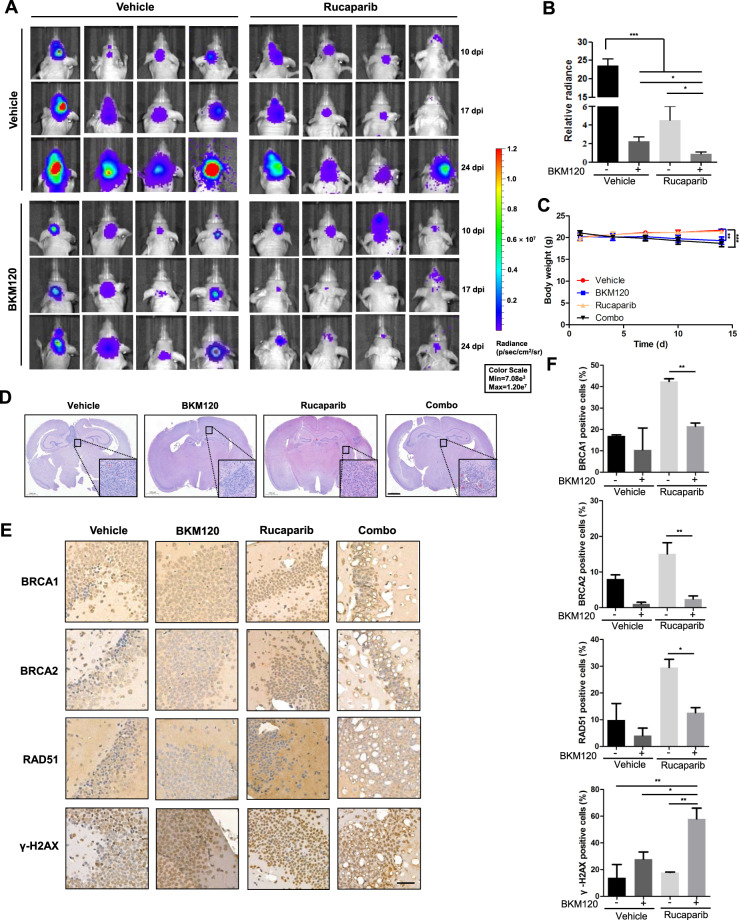
BKM120/rucaparib combination treatment inhibits tumor growth in U87MG nude mouse orthotopic xenograft models. **A**–**F** Mice were inoculated with U87MG-Luc cells. The localization and intensity of luciferase expression were monitored by in vivo bioluminescence imaging (dpi, days post cell injection). Following a single dose of BKM120 (30 mg/kg) or rucaparib (10 mg/kg) or combination treatment of BKM120 and rucaparib for 14 days (*n* = 5), animals were measured by a bioluminescence imaging system. **A** Representative images of mice taken by the bioluminescence imaging system. **B** The relative radiance, which represents tumor size, was determined by the imaging system. **C** Body weights were monitored during the 14 days of treatment. **D** Hematoxylin and eosin-stained section (original magnification ×20) of intracranial U87MG mouse xenografts following BKM120 and/or rucaparib treatments. Scale bar: 1 mm. **E** Representative immunohistochemistry staining for BRCA1/2, γ-H2AX, and RAD51 in tumor tissues of U87MG xenografts. Scale bar: 50 μm. **F** Statistical quantification of (**E**) shows HR repair efficiency and DNA DSB levels in U87MG xenografts with the indicated treatments. Data are presented as the mean ± SD (*n* = 3). *P < 0.05; **P < 0.01; ***P < 0.001.

Finally, we used an U87MG orthotopic nude mouse xenograft model to examine whether combination of the two drugs could exhibit anti-GBM efficacy after passing the blood brain barrier (BBB), which is known as a barrier for water-soluble drugs. U87MG cells stably expressing luciferase (U87MG-Luc) were used to facilitate live imaging of tumor growth. Ten days postinjection of U87MG-Luc cells in the corpus striatum of mice, mice were subjected to drug treatment. After 14 days of treatment, either BKM120 or rucaparib exhibited significant antitumor effects as a single agent. When combined together, tumor burden was further significantly reduced compared to each drug alone (Fig. 6A, B). However, both BKM120 and the combination treatment caused a reduction in body weight, suggesting potential toxicity (Fig. 6C). The brain tissue stained with hematoxylin and eosin showed that U87MG orthotopic xenografts grew in a noninvasive way, and tumors from mice administered combination treatment were smaller than tumors from the other groups (Fig. 6D). Immunohistochemical analysis indicated downregulation of BRCA1/2 and Rad51 and upregulation of γ-H2AX in response to the combination of rucaparib and BKM120 compared to rucaparib alone, consistent with the in vitro results (Fig. 6E, F).

## Discussion

PARPis have exhibited convincing antitumor efficacy on BRCA-deficient ovarian and breast tumors^13^. In GBM, where BRCA1/2 functional mutations/deletions are rare (≤1%)^15^, PARPi merely exerts strong anti-GBM effects in a few cases, such as those with IDH1/2 mutations^16^. However, in most cases, BRCA1/2 can be recruited to damaged DNA for the repair of DSBs, reducing the antitumor efficiency of PARPis^17^. On the other hand, PI3K was reported to stabilize and preserve DSB repair by interacting with the HR complex^18^. Combination therapies with PARPi and PI3Ki have shown considerable promise for the treatment of ovarian cancer^19,20^, triple-negative breast cancer^21,22^ and prostate cancer^23^. In this study, we found that PI3Ki BKM120 induced HR deficiency by downregulating the DNA repair process, sensitizing BRCA-proficient GBM to PARPi.

We demonstrated that combination of the PARPi rucaparib with the PI3Ki BKM120 resulted in synergetic inhibition of GBM cell proliferation. Cell cycle analysis indicated that rucaparib alone arrested the cell cycle in G2/M phase, but when combined with BKM120, the effect was weakened. Meanwhile, apoptosis and DNA damage were most serious in cells treated with the combination. It was reported that PARPi activates the G2/M checkpoint and causes cell cycle arrest in G2/M until DNA damage was repaired^24^. Therefore, BKM120 inhibited DNA damage repair and eventually led to apoptosis, which might be related to the attenuation of the G2/M checkpoint arrest caused by rucaparib. However, this postulation remains to be verified since the above attenuation of G2/M arrest may also be regarded as enhancement of G1 arrest.

We also found that BKM120 combined with rucaparib induced a marked increase in DSBs, one of the most harmful lesions to cells^15,25^. It is well known that the HR pathway plays a major role in the late S and G2 phases to repair DSBs, so we examined whether BKM120 enhanced the effect of rucaparib by regulating HR. The HR reporter assay revealed that HR repair efficiency was reduced after cotreatment of rucaparib and BKM120 compared to rucaparib alone. BKM120 also inhibited the transcription and translation of the HR molecules BRCA1 and BRCA2. Ibrahim et al reported that PI3K inhibition resulted in the compensatory activation of MAPK/ERK pathway, thus phosphorylated and activated ETS (E26 Transformation-specific Sequence) transcription factors to reduce BRCA1/2 transcription through repressing the BRCA1/2 promoter, eventually induced HR repair deficiency in triple-negative breast cancer cells^21^. In addition, RRM2, a downstream effector of BRCA1, was also reduced after rucaparib/BKM120 treatment. Rismussen et al. reported that BRCA1 loss impedes replication fork progression by downregulating RRM2 in GBM cells^17^. Furthermore, BKM120 treatment decreased Rad51 expression and foci formation, which might be attributed to the inactivation of AKT, as AKT inactivation was reported to inhibit Rad51 expression^26,27^. Besides, the ATR-CHK1 pathway is the principal effector of DNA damage and is essential for HR. ATR signaling was also demonstrated to be involved in PARPi resistance^28^. Kumar et al found that PI3K-p110β played a key role in DSB regulation. Knockdown of PIK3CB gene inhibited the recruitment and phosphorylation of ATR, and subsequently the phosphorylation of CHK1, thus increased genomic instability^29^. Therefore, we investigated the effect of rucaparib/BKM120 on ATR and CHK1 and found that BKM120 attenuated phosphorylation of ATR and CHK1, inhibiting HR and sensitizing cells to rucaparib.

Notably, we discovered that BKM120 and rucaparib complemented each other during the DNA damage response. Since tumor resistance is frequently reported to be mediated by activation of the PI3K pathway, targeting PI3K may be a plausible strategy for the treatment of GBM^18^. BKM120 induced an increase in PAR, which was reversed by cotreatment with rucaparib, as shown in both in vitro and in vivo experiments (Fig. 3B and Fig S3). Consistent with our result, BKM120 was reported to upregulate PAR as well as H2AX in BRCA1-related breast cancer^21^. Suppression of the PI3K pathway was accompanied by cleaved PARP upregulation and an increase in PARP activity (increased PAR levels), suggesting that cells undergoing PI3K suppression may be recovered by PARP-dependent DNA repair. GBM cells widely express PARP-1; therefore, targeting PARP-1 might represent an appropriate strategy for overcoming apoptotic resistance^30,31^.

We used three types of subcutaneous and orthotopic xenograft models^32,33^ to evaluate the in vivo anti-GBM effect of the BKM120/rucaparib combination. Consistent with the in vitro studies, the combination obviously enhanced the antitumor efficacy in all U87MG xenograft models compared to each drug alone. Western blotting and IHC analysis of tumor tissues indicated that rucaparib increased expression of the HR repair effectors BRCA1/2 and Rad51, while these molecules were reduced by the combination, suggesting that BKM120 sensitizes tumor tissues to the effect of rucaparib by suppressing HR repair in vivo. Failure to cross the blood–brain barrier (BBB) is the bane of drug development for cerebral disease. Similar to many other drugs, rucaparib has been proven to be a substrate of efflux transporters in brain endothelial cells; thus, the ability of rucaparib itself to pass through the BBB is limited^34,35^. Recent studies have shown that PI3Ki can increase drug accumulation in cells by inhibiting the expression of efflux transporter protein P-gp and/or BCRP^36,37^. We found that rucaparib increased the expression of BCRP and P-gp in brain tissues, which could be suppressed by cotreatment with BKM120. And BKM120 alone could downregulate the two transporters as well, especially BCRP (Fig. S3). Therefore, in addition to inhibiting HR, we speculated that BKM120 might sensitize the in vivo antitumor effect of rucaparib by increasing its effective concentration in the tumor area. However, it should be noted that the body weight of nude mice treated with the combination was slightly lower than those treated with BKM120 or rucaparib alone, which might be due to combined cytotoxicity. Therefore, appropriate doses of the two compounds should be characterized in early clinical trials, and sequential therapy might be considered to reduce toxicity.

Taken together, we demonstrated that combined administration of the PI3Ki BKM120 and the PARPi rucaparib is effective at inhibiting GBM tumor growth both in vitro and in vivo. Cotreatment with BKM120 and rucaparib synergistically induces apoptosis and DNA damage in GBM cells. PI3K inhibition by BKM120 suppresses HR, conferring sensitivity to rucaparib. Our study suggests that combined blockade of PI3K and PARP might be a promising therapeutic approach for GBM, but further investigations are needed to confirm these findings.

## Materials and methods

### Cell culture and reagents

U251 and U87MG cell lines were purchased from Cell Resource Center, Peking Union Medical College (Beijing, China). Cells were cultured in RPMI 1640 (for U251) or DMEM (for U87MG) supplemented with 10% FBS and 1:100 penicillin/streptomycin (Invitrogen, Carlsbad, CA, USA) at 37 °C in 5% CO_2_. Cell lines were authenticated by STR profiling and confirmed to be without mycoplasma contamination.

BKM120 and rucaparib were purchased from Selleck (Danvers, MA, USA). MTT reagent was purchased from Amresco (Solon, OH, USA). The Annexin V-FITC/PI Apoptosis Detection Kit was purchased from BD Biosciences (San Jose, CA, USA). TRIzol reagent was purchased from Life Technologies (Carlsbad, CA, USA). Star Script II First-strand cDNA Synthesis Mix and 2× RealStar Green Fast Mixture were purchased from GenStar (Beijing, China). Antibodies against RAD51 (#385533), BRCA-1 (#342823), BRCA-2 (#160133), ATR (#161471), CHK1 (#380200) were purchased from zen-bio (Chengdu, China), caspase-3 (#9662), BRCA-1 (#9010), BRCA-2 (#10741), PARP (#9532), p-ATR (#2853), p-CHK1 (#2347), RRM2 (#65939), PAR (#83732) ABCB1 (#13978), ABCG2 (#42078), γ-H2AX (#80312), and Ki67 (#9449) antibodies were purchased from Cell Signaling Technology. Alexa Fluor 488 (#ab150113)- and Alexa Fluor 594 (#ab150080)-conjugated secondary antibodies were purchased from Abcam. pDR-GFP, pCBASce-I plasmids, and pEGFP-C1 were kindly provided by Dr. François X. Claret (MD Anderson Cancer Center, Houston, TX).

### Cell proliferation assay

Cell proliferation was measured by MTT assay as we previously described^38^. The cells were seeded at a density of 5 × 10^3^ cells/ml in a volume of 200 μl/well in 96-well plates. The next day, cells were treated with DMSO or a series of concentrations of BKM120 or rucaparib. Four days later, 20 μl of MTT (5 mg/ml) was added to each well. After further incubation for 4 h at 37 °C, the absorbance at 490 nm was measured using an iMark microplate reader (BIO-RAD, Hercules, CA, USA).

### Drug combination analysis

First, the MTT assay was performed as described in 2.2. The half-maximal inhibitory concentration (IC_50_) values were acquired from dose-response curves utilizing GraphPad Prism 7 software. Then, drug synergy was analyzed based on Chou and Talalay’s equation^14^. Briefly, three cell lines were treated for 4 days with BKM120 or/and rucaparib at a constant concentration. Data obtained from the growth inhibitory experiments were analyzed by CalcuSyn software to determine the drug combination effect. Combination indexes (CI) were then calculated, with CI < 1, CI = 1 and CI > 1 indicating synergism, additivity, and antagonism, respectively.

### Colony formation assay

The colony formation assay was performed as previously described^39^. U251 and U87MG cells were seeded into 24-well plates at 500 cells/well and then treated with BKM120 and/or rucaparib or vehicle control (DMSO) the next day. Cell culture medium containing drug or vehicle was changed every three days. After incubation for 10 days, colonies were fixed and stained with 0.25% crystal violet (Sigma-Aldrich, St. Louis, MO, USA) for visualization. Colonies with 50 or more cells were counted using ImageJ software.

### Flow cytometry

Detection of apoptosis was performed using an Annexin V-FITC/PI Apoptosis Detection Kit (BD Biosciences) as previously described^40^. Briefly, U251 and U87MG cells were seeded into 6-well plates at a density of 40% confluence. The next day, cells were exposed to BKM120 and/or rucaparib. After 24/72 h of treatment, cells were collected and suspended in binding buffer containing Annexin V-FITC. The cell suspension was further incubated with propidium iodide (PI) for 15 min. Apoptosis was detected by FACS Verse Flow Cytometry (BD Biosciences) and quantified using Flow Jo Software (Tristar, Ashland, OR, USA). The percentage of cells in the upper right (representing late apoptotic cells) and lower right quadrants (representing early apoptotic cells) were summed to obtain the percentage of apoptotic cells.

For analysis of cell cycle distribution, cells were seeded into 6-well plates and treated with the indicated drugs on the following day as reported in our previous study^41^. After 24/48/72 h of incubation, cells were collected, suspended in PBS, and fixed in 75% ethanol at 4 °C overnight. Next, cells were washed and resuspended in PBS containing 50 μg/ml PI and 100 μg/ml RNase (Solabio, Beijing, China). All samples were analyzed on a FACS Verse Flow Cytometer (BD Biosciences).

### Alkaline comet assay

After treatment with BKM120 or/and rucaparib for 12/24 h, U251 and U87MG cells were collected and subjected to alkaline comet assay for detection of both SSBs and DSBs as we described previously^42^. The cells (1 × 10^5^/ml) were combined with molten LMAgarose at a ratio of 1:10 (v/v), and 50 µl of cell suspension was immediately added onto comet slides. The slides were then incubated at 4 °C for 10 min, immersed in lysis solution for 30 min, and in alkaline unwinding solution for 20 min in the dark. Following electrophoresis, the cells were stained with ethidium bromide (EB). Approximately 200 individual cell images of each group were analyzed using CASP software (CaspLab, Wroclaw, Poland). Tail DNA (percentage of DNA in the tail) was calculated from at least 50 cells of each group as an indicator of DNA damage.

### Western blotting

Western blot analysis was performed as described in a previous study^43^. U251 and U87MG cells were treated with BKM120 and/or rucaparib for 48 h. Then, cells were harvested and lysed in RIPA buffer supplemented with 1% phosphorylation inhibitors and 1% protease. Total protein (50 µg) for each sample was loaded onto an SDS-PAGE gel. After electrophoresis, proteins were transferred onto polyvinylidene fluoride membranes (BIO-RAD). After blocking in 5% nonfat dried milk, membranes were incubated with primary antibodies overnight at 4 °C and then washed and incubated with HRP-conjugated secondary antibodies for 1 h. Expression of target protein was visualized using an ECL detection kit (Thermo Fisher Scientific, Inc., Carlsbad, CA, USA).

### Immunofluorescence staining

Immunofluorescence staining was performed as described in our previous report^42^. U251 and U87MG cells were cultured on coverslips in 24-well plates in the respective media containing inhibitors for 12/24 h. Then, cells were fixed with 4% paraformaldehyde, diluted with PBS, and permeabilized with 0.5% Triton X-100 buffer for 20 min. After mounting, coverslips were immunostained with mouse monoclonal anti-γ-H2AX or rabbit polyclonal anti-RAD51 overnight at 4 °C. The next day, coverslips were washed and incubated using the respective secondary antibody (1:50) at room temperature for 1 h. Nuclei were counterstained using DAPI (Solabio, Beijing, China). An Olympus FV1000 scanning confocal microscope (Toyko, Japan) was used to capture images. The γ-H2AX and RAD51 foci were counted from at least 50 cells per sample.

### Homologous recombination assay

HR efficiency was evaluated as reported in a previous study^44^. U251 and U87MG cells were seeded into 6-well plates and cultured overnight. The HR repair reporter substrate pDR-GFP plasmid and the pCBASceI plasmid were transfected into cells using Lipofectamine 3000 (Thermo Fisher, Illkirch, France). GFP-expressing plasmid (pEGFP-C1) was used as a transfection efficiency control. Cells were treated with BKM120 and/or rucaparib for 48 h. Then, cells were harvested and resuspended in ice-cold PBS, and GFP-positive cells were detected using a FACS Verse flow cytometer (BD Biosciences).

### Quantitative real-time PCR

qRT-PCR analysis was performed as previously reported^45^. U251 and U87MG cells were treated with BKM120 and/or rucaparib for 48 h. Total RNA was isolated from cells using TRIzol according to the manufacturer’s protocol. cDNA was synthesized from 1 μg of total RNA using StarScript II First-strand cDNA Synthesis Mix (Genstar), and RT-PCR was performed with aliquots of cDNA samples mixed with 2× RealStar Green Fast Mixture. Results are expressed as the fold change calculated by the ΔΔC_t_ method relative to the control sample. The ribosomal subunit 18 S was used as an internal normalization control. Sequences of the PCR primers were as follows: BRCA1 Fw 5′-GAACGGGCTTGGAAGAAAAT-3′ and Rv 5′-GTTTCACTCTCACACCCAGA-3′. BRCA2 Fw 5’-CAGGTAGACAGCAGCAAGCA-3’; Rv 5’-AAGCCCCTAAACCCCACTTC-3’. RAD51 Fw 5’-CAGATGCAGCTTGAAGCAAA-3’; Rv 5′-TTCTTCACATCGTTGGCATT-3′. 18 S rRNA Fw 5′- CAGCCACCCGAGATTGAGCA-3′; Rv 5′-TAGTAGCGACGGGCGGTGTG-3′.

### U87MG orthotopic zebrafish model

Xenotransplantation of human glioblastoma U87MG cells and proliferation assessment were performed as described in a previous study^33^. Wild type AB zebrafish (*Daniorerio*) were maintained and reared on a 14 h light-10 h dark cycle at 28 °C in a controlled multitank recirculating system. Embryos were collected and incubated in reconstituted water (60 µg/ml sea salt in RO water with 1 ppm methylene blue). At 48 h postfertilization, embryos were anesthetized using 1.2 mM tricaine and moved onto a modified agarose gel mold for tumor cell microinjection. A total of 50–100 U87MG-RFP cells suspended in 5 nl of serum-free culture medium were injected into the brains of zebrafish larvae via a pneumatic pico-pump injector. After cell implantation, injected embryos were screened and separately transferred to a 48-well plate containing drugs in 2 ml of E3 media and incubated at 32 °C for 4 days. Xenografts were observed with an Olympus (IX71) inverted microscope every 2 days. The fluorescence intensity of xenografts was quantified using ImageJ software.

### U87MG heterotopic nude mouse model

All animal experiments were performed in accordance with the National Institutes of Health Guide for the Care and Use of Laboratory Animals (Ethical approval No.: IACUC-DWZX-2020-753. Date: 25 August 2020 to 25 October 2020). To generate a murine subcutaneous tumor model, U87MG cells (1 × 10^7^ cells per mouse) were injected subcutaneously into the right lateral flank of 4- to 5-week-old male BALB/C nude mice (Vital River Laboratory Animal Technology Company, Beijing, China). When tumors reached a volume of 800–1000 mm^3^, tumor masses were divided into 2 × 2 × 2 mm^3^ masses and implanted into the right flank of 28 BALB/c nude mice. Tumors were allowed to grow to a volume of 100 mm^3^, and then the animals were randomly divided into four groups. Each group of 7 mice was treated with vehicle, BKM120 (15 mg/kg, po.), rucaparib (4 mg/kg, ip.), or BKM120 and rucaparib (with the same doses as a single agent). Tumor size was measured every three days until the endpoint, and the volume was calculated using the formula (length × width^2^)/2. At the end of the 16 days, the mice were sacrificed by using excessive pentobarbital sodium, and then the tumors were removed. Half of the tumor tissues were formalin-fixed and paraffin-embedded for histological analysis, and the other half of tumors were snap-frozen in liquid nitrogen for western blotting.

### U87MG orthotopic mouse model

To generate a murine intracranial tumor model, U87MG-Luc cells (2.0 × 10^5^ cells per mouse) were injected into the right striatum (1 mm lateral to bregma, 0.8 mm anterior to bregma, and 3 mm deep from the dura) of 4- to 5-week-old male BALB/C nude mice. Nine days later (Day 10), animals were measured using an IVIS luminescent imaging system (IVIS Spectrum, PerkinElmer) and randomly divided into four groups. The respective groups of mice were treated as follows: vehicle, BKM-120 (30 mg/kg), rucaparib (10 mg/kg), BKM-120, and rucaparib (with the same doses as each single agent). After treatment for 7 and 14 days, animals were examined by luminescence imaging. At the end of the 14 days, the mice were sacrificed by using excessive pentobarbital sodium. Data were analyzed using in vivo imaging software.

### Examination of the compounds in brain tissues of non-tumor mouse

We used non-tumor mouse to evaluate the effect of rucaparib and BKM120 alone or in combination on PARylation and efflux transporters in the brain tissue, to verify that the 2 compounds passed through BBB. Four-five weeks BALB/C nude mice were treated as follows: vehicle, BKM120 (30 mg/kg), rucaparib (10 mg/kg), or BKM120/rucaparib combination (with the same doses as each single agent) for 3 days. Then, the mice were killed by using excessive pentobarbital sodium and the brain tissue was removed. Half of the tissue was formalin-fixed and paraffin-embedded for histological analysis, and the other half was snap-frozen in liquid nitrogen for western blotting.

### H&E and immunohistochemical (IHC) staining

H&E and IHC staining were performed as previously reported^46^ with a small modification. H&E staining was used to detect pathological changes. For histological analysis, formalin-fixed, paraffin-embedded tumors were sectioned, and slides were deparaffinized using xylenes (Thermo Fisher Scientific Inc.). Endogenous peroxidases were quenched with 3% hydrogen peroxide in methanol. Staining was performed using antibodies against BRCA1 (1: 100), BRCA2 (1: 100), RAD51 (1: 50), γ-H2AX (1: 50), P-gp (1: 200), BCRP (1: 200), PAR (1: 250), or Ki67 (1: 500). Counterstaining was performed using Mayer’s hematoxylin (Dako, Glostrup, Denmark). Images were observed under an Olympus Cx21 microscope, scanned with a high-resolution digital slide scanner (Pannoramic 250, 3DHistech), and quantified using ImageJ software.

### Statistical analysis

All results were repeated in triplicate unless otherwise stated, and quantitative data are expressed as the mean ± standard deviation (SD). Statistically significant differences between pairs of mean values were determined by Student’s *t*-test. Statistical significance was defined as a *P*-values < 0.05.

## Supplementary information

Suppl figure legends

Suppl figure 1

Suppl figure 2

Suppl figure 3

## Data Availability

The datasets used and/or analyzed during the current study are available from the corresponding author on reasonable request.
