# Supplementary material for: BKM120 sensitizes glioblastoma to the PARP inhibitor rucaparib by suppressing homologous recombination repair
Source: Cell Death Dis. 2021 May 26;12(6):546. doi: 10.1038/s41419-021-03805-6 (PMC8150626; doi:10.1038/s41419-021-03805-6)
Supplement: Supplementary file 1 — Suppl figure legends [file 41419_2021_3805_MOESM1_ESM.docx]

**Fig. S1: Effect of BKM120 and rucaparib as single agents or in combination on cell cycle distribution after treatment for 72 h.** (A) Cells were treated with BKM120 and/or rucaparib for 72 h. After PI staining, flow cytometry analysis was performed to determine the cell cycle distribution. (B) Cell cycle distribution of the cells exposed to the single drugs or combination are represented as stacked columns. Statistical analysis of G2/M phase in each group. Data are presented as the mean ± SD (n=3). *P < 0.05; **P < 0.01; ***P < 0.001.

**Fig. S2: Effect of BKM120 and rucaparib as single agents or in combination on cell apoptosis after treatment for 24 h.** U251 and U87MG cells were treated with BKM120 and rucaparib as single agents or in combination. (A) The percentage of apoptotic cells was determined by Annexin V/PI staining after treatment for 24 h. (B) FACS quantification of the respective cell population including Annexin V+/propidium iodide− early apoptotic cells, Annexin V+/propidium iodide+ late apoptotic cells and Annexin V-/propidium iodide+ necrotic cells. Data are presented as the mean ± SD (n=3). *P < 0.05; **P < 0.01; ***P < 0.001.

**Fig. S3:** **Effect of BKM120 and rucaparib as single agents or in combination on PAR and efflux transporter P-gp and BCRP in non-tumor mouse.** Non-tumor nude mouse were treated with vehicle, BKM120 (15 mg/kg), rucaparib (4 mg/kg) or the BKM120/rucaparib combination for 3 days. After sacrificing mice, the brain tissue was removed. (A) Western blotting analysis of PAR, P-gp and BCRP in harvested brain tissue. (B) Representative immunohistochemistry staining for PAR, P-gp and BCRP. Scale bar: 50 μm. Immunohistochemistry statistical quantification was shown in (C). Data are presented as the mean ± SD (n=3). *P < 0.05.
