# Supplementary figures and images for: BKM120 sensitizes glioblastoma to the PARP inhibitor rucaparib by suppressing homologous recombination repair
Source: Cell Death Dis. 2021 May 26;12(6):546. doi: 10.1038/s41419-021-03805-6 (PMC8150626; doi:10.1038/s41419-021-03805-6)

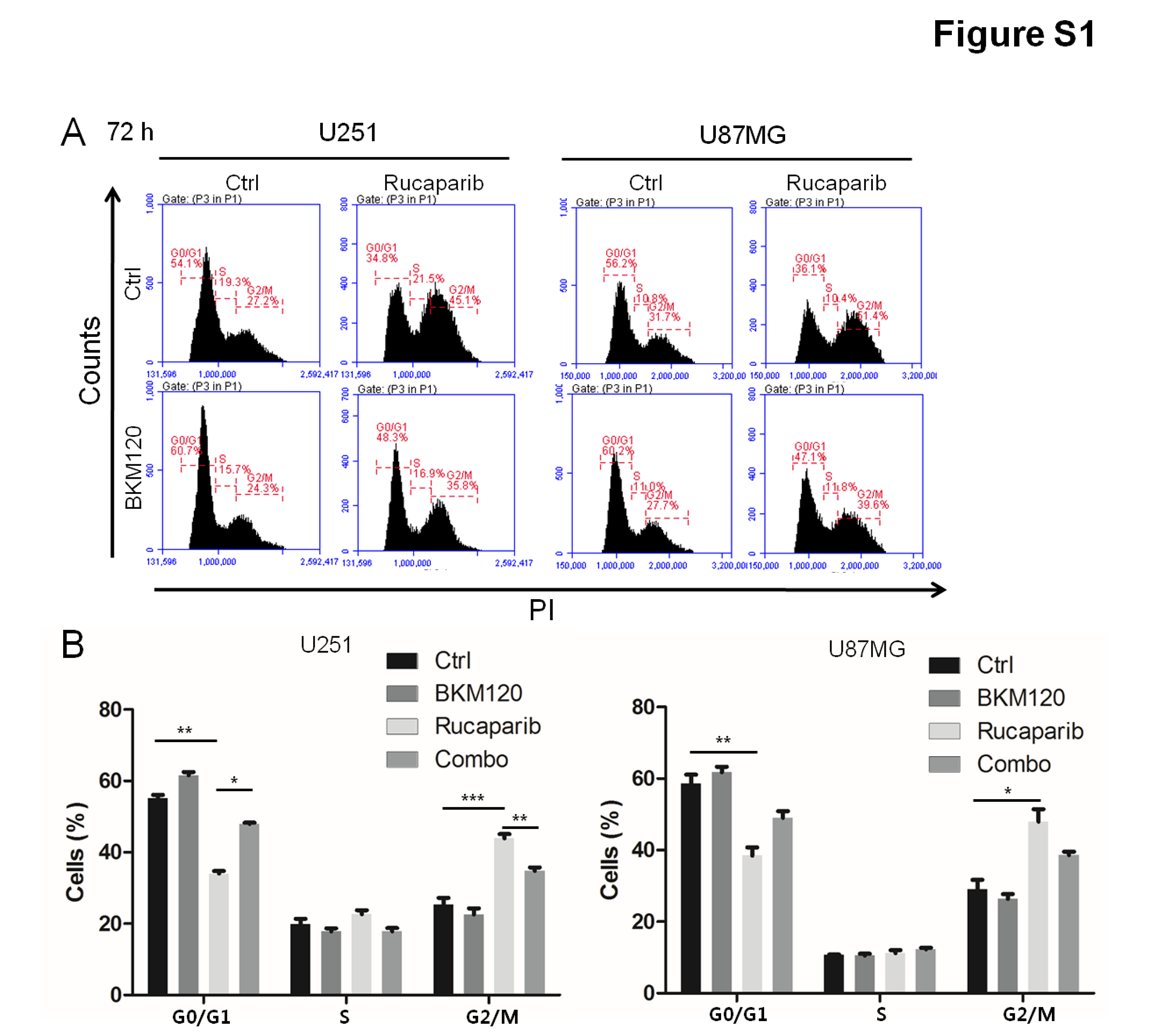

Supplement: Supplementary file 2 — Suppl figure 1 [file 41419_2021_3805_MOESM2_ESM.tif]

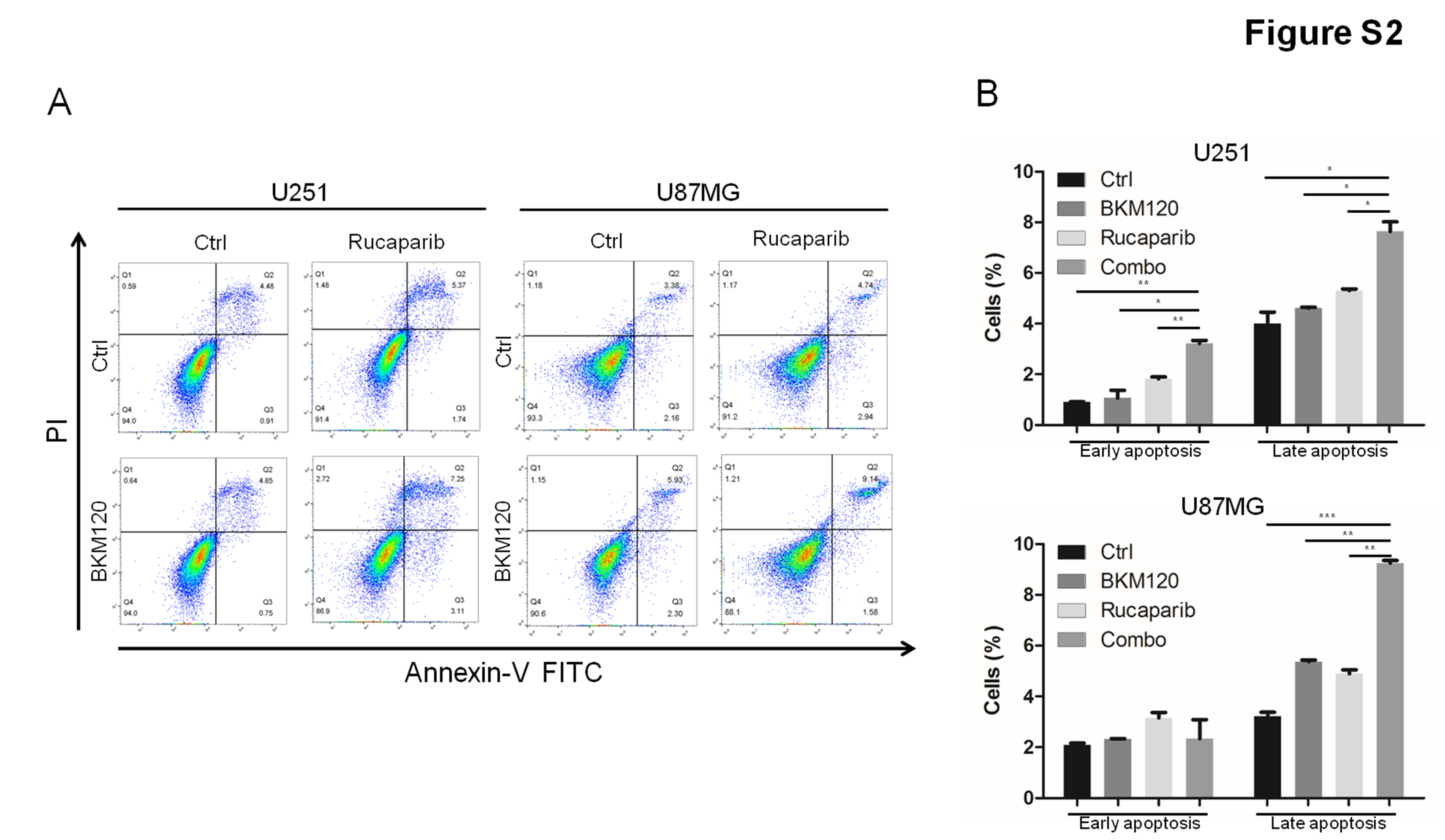

Supplement: Supplementary file 3 — Suppl figure 2 [file 41419_2021_3805_MOESM3_ESM.tif]

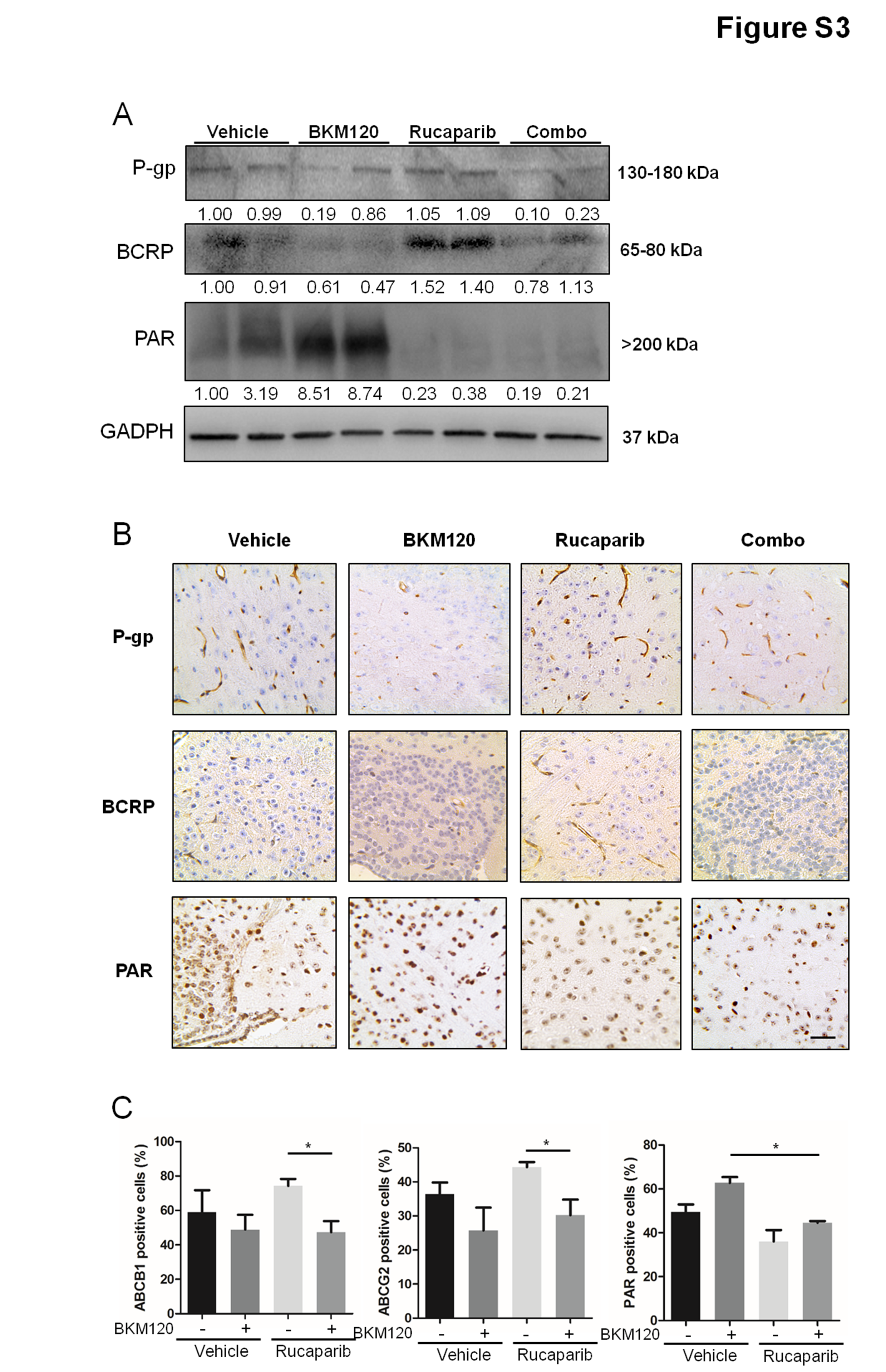

Supplement: Supplementary file 4 — Suppl figure 3 [file 41419_2021_3805_MOESM4_ESM.tif]
